# A Medical Examination at the Surgeons' Company in 1789

**Published:** 1903-08-29

**Authors:** 


### A MEDICAL EXAMINATION AT THE SURGEONS' COMPANY IN 1789.

Mr. Peachey, in his life of William Savory, of Brightwalton, gives the following account of the examination to which Savory was subjected before he was admitted to the freedom of the Surgeons' Company.

1789, May 7. I was examined at Surgeons' Hall, and had the honour conferred upon me to be a member of their body. This Hall is a large pile of buildings in the Old Bailey Street. We were called into the room and examined one at a time. About a fortnight before I was examined I went to the Hall and entered my name in a book and paid 2s. 6d. When I entered the room I was a little timid by seeing the surgeons; however, I recovered of that dread, and answered every question they asked me. The table they sit round is in the form of a semi-circle: the Master sits in the middle, and the two wardens, one each side, three or four surgeons each side of the wardens, and the surgeons examine the pupils by turns. The Master asked me where I received my instruction. I told him at St. Thomas's Hospital, and attended lectures under Mr. Cline. I was then ordered to Mr. Serjeant Surgeon Pile to be examined:—(*Q.* = question. *A.* = answer).

*Q.*—What are the common Integuments?

*A.*—The cutis, cuticle and rete mucosum.

*Q.*—What are the arteries?

*A.*—Vessels which carry the blood from the heart.

*Q.*—What comes first to view after the common integuments are taken off the abdomen?

*A.*—The linea alba.

*Q.*—How does the urine enter the bladder?

*A.*—By the ureters obliquely.

*Q.*—What are the parts in the thorax?

*A.*—The heart, lungs, pleuræ, etc.

Now I have asked you a few questions in anatomy, I shall ask you a few in surgery.

*Q.*—Where would you cut off a person's leg?

*A.*—If below the knee I would amputate about four or five inches below the extremity of the patella.

*Q.*—But where would you make your first incision?

*A.*—A little below to allow for the excess of contraction.

*Q.*—Where would you apply the tourniquet, and how would you stop the hæmorrhage after the limb is amputated?

*A.*—I would apply the tourniquet on the middle of the thigh, because there the artery is nearer the bone; and I would stop the blood with a needle and ligature, and after that I would keep the skin forward with a bandage, and apply dry lint and adhesive plaster.

*Q.*—Which side would you stand on to amputate the leg?

*A.*—On the inside, because of taking off the fibula first.

*Q.*—Suppose your patient was weak and low after the operation, what would you give him?

*A.*—I would give him bark.

*Q.*—Suppose you had a stone in the urethra, how would you extract it?

*A.*—If it was impossible for me to get it out without making an incision, I would pull the prepuce over the glans, and make an incision the length of the stone through the teguments.

It cost me £13 5s: the diploma cost the 5s. We were all called in afterwards, and took our oaths and received a little pamphlet containing rules and orders. The next day we received our diplomas.

